# Molecular Mechanisms Linking Risk Factors to Cholangiocarcinoma Development

**DOI:** 10.3390/cancers14061442

**Published:** 2022-03-11

**Authors:** Ludovica Ceci, Tianhao Zhou, Ilaria Lenci, Vik Meadows, Lindsey Kennedy, Ping Li, Burcin Ekser, Martina Milana, Wenjun Zhang, Chaodong Wu, Keisaku Sato, Sanjukta Chakraborty, Shannon S. Glaser, Heather Francis, Gianfranco Alpini, Leonardo Baiocchi

**Affiliations:** 1 Hepatology and Gastroenterology Division, Department of Medicine, Indiana University, Indianapolis, IN 46202, USA; lceci@iu.edu (L.C.); zhouv@iu.edu (T.Z.); vikmead@iu.edu (V.M.); linkenn@iu.edu (L.K.); keisato@iu.edu (K.S.); heafranc@iu.edu (H.F.); 2 Unit of Hepatology, Tor Vergata University, 00133 Rome, Italy; ilaria.lenci@uniroma2.it (I.L.); martinamilana@gmail.com (M.M.); 3 Department of Research, Richard L. Roudebush VA Medical Center, Indianapolis, IN 46202, USA; 4 Department of Surgery, Division of Transplant Surgery, Indiana University, Indianapolis, IN 46202, USA; pili@iupui.edu (P.L.); bekser@iupui.edu (B.E.); wenzhang@iu.edu (W.Z.); 5 Department of Nutrition, Texas A&M University, College Station, TX 77843, USA; cdwu@tamu.edu; 6 Department of Medical Physiology, Texas A&M University College of Medicine, Bryan, TX 77807, USA; schakraborty@tamu.edu (S.C.); sglaser@tamu.edu (S.S.G.)

**Keywords:** cholangiocarcinoma, risk factor, liver fluke, primary sclerosing cholangitis, bile duct cysts, cirrhosis

## Abstract

**Simple Summary:**

Cholangiocarcinoma is the second most frequent primary liver tumor in humans. In this review, we report current findings on the mechanisms linking well-known risk factors to this cancer. These pathways may be in part involved in risk factors unrelated to cholangiocarcinoma forms.

**Abstract:**

The poor prognosis of cholangiocarcinoma in humans is related to several factors, such as (i) the heterogeneity of the disease, (ii) the late onset of symptoms and (iii) the limited comprehension of the carcinogenic pathways determining neoplastic changes, which all limit the pursuit of appropriate treatment. Several risk factors have been recognized, including different infective, immune-mediated, and dysmorphogenic disorders of the biliary tree. In this review, we report the details of possible mechanisms that lead a specific premalignant pathological condition to become cholangiocarcinoma. For instance, during liver fluke infection, factors secreted from the worms may play a major role in pathogenesis. In primary sclerosing cholangitis, deregulation of histamine and bile-acid signaling may determine important changes in cellular pathways. The study of these molecular events may also shed some light on the pathogenesis of sporadic (unrelated to risk factors) forms of cholangiocarcinoma, which represent the majority (nearly 75%) of cases.

## 1. Introduction

Cholangiocarcinoma (CCA) represents the most frequent cancer encountered in the biliary system, and, according to its anatomical location, it has been distinguished in intrahepatic (i-CCA), perihilar (p-CCA) and distal (d-CCA) forms [1]. Even though CCA may be considered a rare disease, with a global incidence of 0.3–6 per 100.000/year, tumor burden may vary according to the geographical distribution of specific risk or genetic factors [2]. CCA mortality has been increasing globally in recent years, accounting for ≤2 out of 100,000 deaths, closely reflecting the prevalence of this malignancy, since therapeutic strategies are generally unsatisfactory [3]. Obstacles in achieving an effective cure for CCA are related to the biological heterogeneity of the disease, the lack of consistent preclinical models and, since its rarity, difficulties in conducting large clinical trials [4]. Even if improved therapeutic results are being achieved for selected patients using targeted therapy, such as pemigatinib [5], an incomplete comprehension of the molecular mechanisms at the base of carcinogenic transformations of biliary epithelia contributes to an unclear horizon for a definitive cure. Moreover, metabolomic analysis of human CCA serum, despite evidencing peculiar changes in lipid profile, is not able to differentiate cancer from other biliary diseases [6].

The identification of the carcinogenic mechanisms that lead well-recognized premalignant conditions to become CCA may have importance in this setting. In this review, we analyze the specific risk factors for CCA development to better understand the possible molecular events leading to cancer.

## 2. CCA Risk Factors

Several CCA risk factors have been identified in humans [7,8]; however, at least in the Western world, the majority of CCAs arise in subjects without a recognizable predisposing condition for this malignancy [9]. CCA risk factors may be traditionally classified between those with a well-established association with regards to the development of the tumor and those with a potential or possible (epidemiologically suggested) role in tumorigenesis [8]. Their distribution among different countries and their general prevalence may change widely, and a preferential link with i-CCA or d-CCA may be observed. In the following review article, we report the most relevant studies according to their epidemiological association with CCA.

### 2.1. Risk Factors Closely Associated with CCA

Among the most important recognized risk factors for CCA development are parasitic, dysmorphogenic and inflammatory afflictions of the biliary tract. In addition, toxic substances, such as thorotrast or 1,2-dichloropropane, have a relevant oncogenic effect on the biliary tree [10,11]. Below we address common risk factors associated with CCA.

#### 2.1.1. Liver Flukes

Approximately 18 million people in the world are infected by the liver flukes *Opisthorchis viverrini* and *Clonorchis sinensis*, with an endemic distribution in eastern Asia and Europe, including countries such as China, Thailand, Laos, Korea, Russia and others [12]. The definite host (a fish-eating mammal) releases eggs in its stool. The second step requires the presence of water snails, where miracidia transform in cercariae. The latter is able to encyst into fish scales and muscle, and transmits infection into the final host (i.e., humans) when this food is consumed uncooked [13,14]. Therefore, it is clear that several conditions must be present to maintain the flukes’ life cycle and to acquire an infection, including (i) adequate environmental conditions for the presence of the two intermediate hosts (snail and fish) and (ii) the desire to consume raw fish. The majority of infected humans are asymptomatic; however, severe symptoms may develop when the infection lingers for several years, and may drive cholangitis, cholestasis and finally tumorigenic transformation of the biliary epithelia [15]. Therapy for liver-fluke infection is mainly based on the use of anti-helminthic drugs (praziquantel); however, treatment of infected humans may be limited by socio-economic variables and the late onset of symptoms.

#### 2.1.2. Primary Sclerosing Cholangitis

Another important risk factor for CCA development in Western countries is primary sclerosing cholangitis (PSC). This ductopenic/cholestatic chronic liver disease has an unclear origin, is strongly associated with inflammatory bowel diseases and demonstrates characteristics of bile duct fibrotic strictures [16]. The overall incidence of PSC is below 1 per 100.000/year in Western countries, supporting its inclusion in the group of rare diseases [17,18]. Major complications are represented by recurrent cholangitis, liver cirrhosis development and certainly CCA. Clinical management is mainly based on the release of dominant strictures (<1.5 mm diameter in common bile ducts or <1 mm in small bile ducts) by endoscopy or liver transplant [19,20]. Several studies addressed the incidence of CCA in patients with PSC, summarized in a recent review published in 2019 [16]. While several possible biases can be identified by comparing different studies (hospital based, geographically restricted, mainly retrospective and others), some convergence exists on CCA incidence, ranging 0.5–1.5% (PSC patients/year) and there is evidence of an increased risk of CCA development in the first 2 years after PSC diagnosis. Based on this, the American Gastroenterological Association (AGA) suggests CCA surveillance for all adult PSC patients, with a particular emphasis on the first year after PSC diagnosis [21].

#### 2.1.3. Bile Duct Cysts

The presence of bile duct cysts is another potential risk factor for CCA development. This rare condition, usually diagnosed during childhood or in young adults, comprises different macroscopic pictures recapitulated by the Todani classification [22]. An increased risk of CCA (20 to 30-fold increase compared to healthy subjects) has been observed when bile duct cysts are present; however, some forms, such as choledochal cysts (Todani type I) or Caroli intrahepatic dilatation (Todani type IV), seem to predispose individuals to the highest risk of CCA, and this is also correlative to patient age [23]. Importantly, a significant risk for CCA remains even after cyst excision [22].

#### 2.1.4. Hepatolithiasis

An additional major risk hazard for CCA development, whichis also present in non-dysmorphic conditions of the biliary tract, is hepatolithiasis (HL).It consists of the presence of gallstones in the intrahepatic (right and left branches) portions of the biliary tree [24]. The vast majority of this obstructive disease is related to the presence of pigmented stones with an increased prevalence in Asian countries, suggesting liver flukes play a role [25]. However, inflammation, cholestasis or bacterial infection in the biliary tree may also determine HL [26]. In Asian countries, the incidence of HL/CCA is significant, lying between 2.4–13% [27], whereas in Western countries this condition appears to be rare [28]. The optimal treatment for CCA during HL is surgical resection, as is the same for CCAs in patients not affected by this disease. While tumor resection is usually feasible in a larger proportion of HL/CCAs, long–term survival outcomes do not significantly change in comparison with the general population that has this cancer [29].

#### 2.1.5. Thorotrast

Exposure to specific toxic substances has been consistently associated with the onset of CCA. Thorotrast, a contrast medium containing radioactive thorium dioxide, has been employed in radiology since the early 1930s, and was discontinued worldwide in the 1960s for its strong association with liver cancer [8]. Thorium dioxide (that is the radioactive component of thorotrast) has a long decay time (half-life 400 years) and scarcely eliminates from the body, thus exposing subjects to a lifelong increase in cancer risk, specifically in organs with enhanced phagocytic activity (such as the liver) [30]. In a Japanese study, the risk of CCA was increased 303 times in patients exposed to Thorotrast [31]. This observation was also supported by a European (German) study [32]. Other environmental toxins (nitrosamines, vinyl chloride and dioxins) have been linked to the onset of CCA, but convincing evidence has not yet been achieved to fully elucidate their impact [33].

### 2.2. Risk Factors with a Suggested Role in CCA Onset

#### 2.2.1. Liver Cirrhosis and Chronic Viral Hepatitis

Other clinical conditions or behaviors that might play a possible role in increased CCA development have been suggested by epidemiological studies and in several non-biliary liver disorders. Liver cirrhosis, which represents a major risk factor for hepatocellular carcinoma (HCC), is characterized by profound changes in liver architecture as a consequence of inflammatory, proliferative/reactive and profibrotic processes, thus also represents an adequate background for iCCA development [34]. A meta-analysis focusing on iCCA-associated conditions (with data gathered from seven case-control studies; total number of patients = 399.608), identified a 23 fold increase in risk for iCCA in cirrhotic patients [35]. In a further Taiwanese study, based on the evaluation of 5.157 CCA cases, the presence of cirrhosis determined an increased 8-fold and 4-fold risk for iCCA and eCCA, respectively [36]. In parallel with cirrhosis, HBV and HCV infections also determine a predisposition for both HCC and CCA. Regarding CCA, patients infected with these viruses seem to have approximately twice the risk compared to healthy controls [9]; however, differences do exist between Asian and Western populations. In fact, in Asia, CCA seems to be associated with HBV and not with HCV infection [37]; whereas in the U.S., the opposite was observed [38]. The different geographical distribution and prevalence of these two viruses may explain, in part, these controversial results.

#### 2.2.2. Non-Alcoholic Fatty Liver Disease

Among the other causes of chronic liver disorders, non-alcoholic fatty liver disease (NAFLD) shows a significant increase in prevalence and related disability [39].The definition of NAFLD remains generic, comprising a spectrum of liver conditions characterized by steatosis (fat accumulation in >5% of hepatocytes) in the absence of alcohol abuse. With these criteria, the global prevalence of NAFLD accounts for approximately 25%, as confirmed by meta-analytic studies [40,41]. However, a distinction should be made between patients with simple steatosis and those with histologically indicative signs of liver inflammation or even fibrosis and potential cirrhosis, which presents in the following much more severe form: non-alcoholic steatohepatitis (NASH). Progression from NAFLD to NASH, in fact, produces the most severe liver complications, leading to an increased risk for end-stage liver disease and cancer [42]. A meta-analysis, based on six case-control studies and one cohort study, found a pooled odds ratio (OR) for CCA in NAFLD of 1.95 [43]. CCA risk was preferential for i-CCA (OR = 2.22) rather than the extrahepatic CCA (e-CCA= p-CCA and d-CCA) (OR = 1.55) form. In the interpretation of these data, it is imperative to note that NAFLD is frequently associated with obesity and type II diabetes, which were also suggested in other studies to have similar ORs for CCA [44,45]. In this respect, understanding the net effect of these different predisposing factors on CCA onset seems difficult.

#### 2.2.3. Inflammatory Bowel Diseases (IBD)

Other non-liver-related diseases or conditions have been associated with CCA carcinogenesis. Inflammatory bowel diseases (IBD), either ulcerative colitis (UC) or Crohn’s disease (CD), have been linked to an increased risk of CCA. A cohort study conducted in Denmark from 1978 to 2003 demonstrated an increased incidence of CCA in IBD patients compared to a control (7.6 vs. 1.9/100.000/yr) with a prevalent distribution in UC subjects rather than in CD ones (8.2 vs. 4.3/100.000/yr) [46]. A following meta-analysis, including four case-control studies and two cohort studies, reported in IBD patients a relative risk (RR) of 2.6 and 1.5 for i-CCA and e-CCA, respectively [47]. These results, however, should be interpreted with caution given the frequent association of PSC, a known important risk factor for CCA (as reported above), with IBD [48]. In fact, in a more recent report coming from the Scandinavian registry of the 10-year mortality for CCA, in IBD patients it was very low (1‰), and it was more frequent in subjects with PSC, either with UC (43.4‰) or CD (0.4‰) [49].

#### 2.2.4. Lifestyle Factors

**Figure 1:**
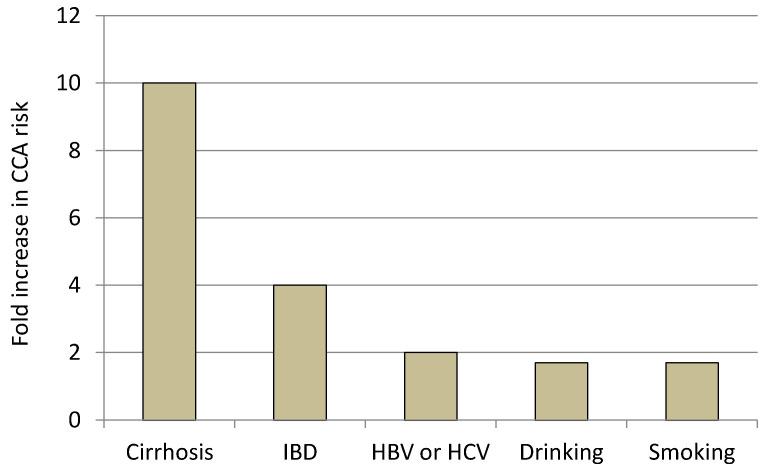
CCA risk in less associated factors. (For reference, in consolidated risk conditions, such as PSC, the hazard is considered more than 10 times higher).

Finally, lifestyle, including drinking and smoking habits, have also been investigated as risk factors for CCA development. Meta-analytic results reported an OR of 1.75 for drinkers (defined in most studies as those consuming >80 g/day) and 1.7 for smokers [9]. The definition of smokers, however, remains unclear due to the strong heterogeneity among different studies. In a more recent Japanese cohort study, smokers (>30 packs/year) had an i-CCA hazard ratio (HR) of 2.2 that was increased by exacerbated drinking (HR = 3.5) [50]. In the same study, smoking alone did not have a significant effect on i-CCA onset. The increase in CCA risk according to less associated hazard factors is depicted in Figure 1.

## 3. Molecular Mechanisms Linking Risk Factors to CCA

Several genetic aberrations and deranged pathways have been observed in CCA, as extensively described in a recent review [4]. Kirsten rat sarcoma (KRAS), isocitrate dehydrogenase (IDH) and fibroblast growth factor/fibroblast growth factor receptor (FGF/FGFR) systems are just some of the pathways that have been observed in CCA growth and progression, contributing to the complex molecular picture of this cancer.

The evaluation and clarification of the pathways altered by a specific risk factor, however, should clarify the general molecular mechanisms at the basis of CCA carcinogenesis. From this perspective, the following sections report current scientific evidence that attempts to identify the mechanisms that lead biliary damage to cause CCA during exposure to the more frequent specific hazards.

### 3.1. From Liver Flukes to CCA

Multiple mechanisms have been suggested to explain CCA onset following liver fluke infections. *O. viverrini*-mediated mechanical damage to the biliary tract is just the first general effect. Other factors, such as the activation of immune processes and parasite-secreted mitogenic molecules, play a role in CCA [51]. For instance, *O. viverrini*-Granulin-1 (Ov-GRN-1), which has a similar sequence of mammalian granulin (an important growth factor likely to be involved in carcinogenesis and tumor spreading) [52], was identified in the biliary epithelia of *O. viverrini*-infected hamsters [53]. Repression of Ov-GRN-1 in RNA-interference experiments demonstrated reduced liver fluke viability and the decreased proliferation of both human cholangiocytes (H69) and CCA (KKU-M214) cell lines [54]. In a separate study, decreased expression of Ov-GRN-1 mRNA was obtained in *O. viverrini* by gene editing techniques [55]. Infection with the edited strain (named ΔOv-GRN-1) in hamsters, attenuated hyperplasia of the biliary cells, as well as peribiliary fibrosis, suggest a possible strategy to interrupt the molecular processes leading from liver fluke infection to CCA. A further mechanism linking Ov-GRN-1 excretion to CCA may involve the process of wound healing. Wound repair machinery has significant similarities with processes activated during carcinogenesis [56]. Fibroblast gene expression profiles, during wound healing, show important similarities with those related to cancer [57]. After ingestion, liver flukes migrate from the intestine to the bile ducts, where chronic injury and repair processes are observed in the biliary epithelium after *O. viverrini* infection. In a study it was demonstrated that Ov-GRN-1 internalization into normal cholangiocytes modified the gene and protein expression pathway, promoting a cellular phenotype that enhanced wound repair [58]. Hence, the ability of *O. viverrini* to cause mechanical damage and simultaneously stimulate Ov-GRN-1-mediated wound healing in the biliary epithelia could promote the transformation of normal cholangiocytes toward a tumorigenic phenotype.

The carcinogenic properties of *C. sinensis* have also been studied and show similarities to *O. viverrini*. Mechanical injury and inflammatory processes also take place during infection by this trematode [59]. Collectively, excreted/secreted products (ESPs) by *C. sinensis* would likely support carcinogenic processes in biliary epithelia. ESPs alter RNA and protein expression, as well as microRNA (miRNA) patterns in CCA cell lines and in mice [60]. Using an in vitro three-dimensional cell model with HuCCT1 (a human CCA line) exposed to ESPs [61], it was shown that the *C. sinensis* molecular factors not only enhanced CCA growth, but also promoted invasiveness. This latter effect was sustained by an increased expression of matrix metalloproteinases (MMPs), and in particular gelatinases and collagenases. In a following study, in the same three-dimensional culture, a ductal plate composed of H69 cells (normal cholangiocytes) was added to investigate the interplay between normal and CCA cells during ESPs exposure [62]. Exposure to ESPs enhanced migration of CCA cells, while those with H69 increased the excretion of interleukin-6 (IL-6) and transforming growth factor-beta1 (TGF-β1). Increased synthesis of these two latter factors enhanced the expression of E-cadherin in HuCCT1 cancer cells, thus supporting their possible epithelial-to-mesenchymal transition (EMT). From this perspective, differential effects are induced by ESPs on normal or neoplastic cholangiocytes. This crosstalk would be able to enhance CCA development and diffusion.

Research was also conducted to assess the effect of *C. sinensis* ESPs on the miRNA profile of a HuCCT1 cell culture [63]. Several miRNAs involved in proliferation and differentiation were altered in response to the infection. In particular, Let-7i, known for its tumor suppressor activity in different cancer types [64], was decreased. This finding suggests the possible restoration of the basal levels of Let-7i as a possible strategy to reduce *C. sinensis*-induced CCA growth and spread.

In conclusion, while both *O. viverrini* and *C. sinensis* are classified as type I carcinogens, several molecular aspects remain to be identified in the progression from biliary infection to CCA. Unfortunately, most experimental studies are conducted in countries where these infections are endemic while other research groups, in different geographic areas, usually neglect studies on fluke infections and carcinogenesis. However, with the robust availability of reliable animal (in hamster) and cellular models, these risk factors should be considered when approaching research that aims to evaluate the progression from biliary damage to CCA.

### 3.2. From PSC to CCA

As previously discussed in the risk factor section, PSC patients are those presenting with the highest risk to develop CCA in Western countries. While the exact molecular mechanisms linking PSC to carcinogenesis are still not completely clarified, increased biliary IL-6 excretion during this disease may play a role. IL-6 production is, in fact, enhanced in PSC by the stimulation of biliary cells by autoantibodies or as part of senescence-associated secretory phenotype (SASP) excreted by senescent cells [65,66]. Interestingly, increased serum levels of IL-6 have been observed in CCA, falling after resection and discriminating this tumor from HCC or colon cancer liver metastasis [67]. Regarding PSC carcinogenic evolution, IL-6 serum levels are similarly enhanced in both PSC and CCA, suggesting this cytokine as a constant accompanying factor in the tumorigenic process [68]. IL-6 stimulates telomerase and miRNA cancer signaling in CCA cells [69]. Moreover, other general mechanisms may include IL-6-mediated activation of survival routes, such as phosphatidylinositol-3 (PI3) kinase, Janus kinase/signal transducer and activator of transcription (JAK/STAT), and p38 mitogen-activated protein (MAP) kinase pathways [70,71,72]. These observations, as well as studies on other tumors, suggest that IL-6 plays an important role in the link between chronic inflammation and cancer, thus supporting this molecule as a possible target for anticancer therapy [73].

Histamine (HA) has been implicated to play a role in both PSC and CCA progression and studies have demonstrated that patients with PSC and CCA have increased HA serum content, therefore, this neurohormone has been targeted as a potential risk factor [74]. In fact, when nu/nu mice were implanted with Mz-ChA-1 cells (a subcutaneous xenograft CCA in vivo model) and treated with exogenous HA, tumor growth exponentially increased compared to controls; however, when HA was removed from Mz-ChA-1 cells using stable shRNA transfection prior to implantation, tumors were remarkably smaller compared to controls, suggesting that HA promotes tumor growth [75]. Since HA interacts with specific H1 and H2 receptors promoting proliferation in biliary epithelia and mast cell infiltration in PSC and CCA, the possible role of this neurohormone in these two pathological conditions was comparatively examined [76]. Using *Mdr2^−/−^* mice (a PSC model) and xenograft tumors derived from Mz-ChA-1 cells implanted into nu/nu mice, blockage of H1 and H2 receptors was attempted with the corresponding inhibitors mepyramine and ranitidine. Inhibition of H1 or H2 HRs significantly attenuated biliary proliferation, inflammation and fibrogenesis in the PSC model, while the same strategy in the CCA model nearly completely inhibited tumor growth, angiogenesis and epithelial-to-mesenchymal transition [76]. Taken together, these findings suggest that HA and downstream-related pathways may have an important role in the pathogenesis of both PSC and CCA. In addition, stimulation by this neurohormone seems similar during these two biliary diseases, suggesting constant HA-signaling activation as a possible risk factor for promoting PSC injury to carcinogenesis in the biliary epithelia.

Another comparative analysis between PSC and CCA human serum samples found possible shared or divergent miRNA pathways [77]. Using this approach, two distinct miRNAs were identified, distinguishing PSC from PSC+CCA, miR-222 and miR-483-5p, respectively, suggesting a possible role for this molecule in the progression from PSC to CCA. In this respect, miR-483-5p overexpression has been found to support tumorigenic processes in other organs, but data on CCA are lacking [78]. On the other hand, data on miR-222 seems less clear. A previous study documented decreased expression of this miRNA in CCA tissue, suggesting its enhanced activity as a possible factor suppressing growth of this cancer [79,80]. This apparent controversy between serum and tissue samples could warrant further studies in the future, including into other miRNAs.

**Figure 2:**
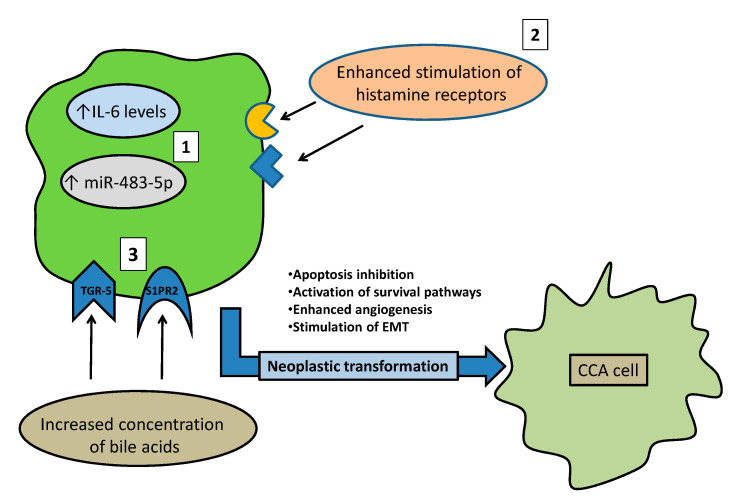
Possible factors leading from primary sclerosing cholangitis (PSC) to cholangiocarcinoma (CCA). The characteristic enhanced: (1) levels of IL-6 and miR-483-5p; (2) stimulation of histamine or histamine receptors; (3) concentration of bile acids (stimulating the specific TGR-5 and S1PR2 receptors) observed in PSC. They are all possible actors in promoting carcinogenesis.

Finally, the increased concentration of bile acids (BAs) in the biliary tract during PSC may have a role in carcinogenesis [81]. BAs (in particular conjugated BAs) are known as important stimulators of CCA growth and spread. In parallel with this, BAs also inhibit apoptosis of biliary cancer cells. These effects are obtained by BA activation of the nuclear factorkappa-light-chain-enhancer of activated B cells (NF-κB) pathway, and stimulation of the Takeda G protein-coupled receptor 5 (TGR-5) and sphingosine-1-phosphate receptor 2 (S1PR2) receptors [82,83,84]. Upon receptor stimulation, ERK 1/2 and AKT pathways sustain the pro-oncogenic processes. From this perspective, it is possible to hypothesize that pharmacological modulation or changes to BAs pool/receptors in cholangiocytes may serve as a possible cure or prevention of CCA in PSC. Possible molecular mechanisms leading PSC to cause CCA are depicted in Figure 2.

### 3.3. From Bile Duct Cysts to CCA

The molecular mechanisms at the base of Type I (Todani classification) choledochal cysts formation are largely unanswered. Similarly, the reasons for the possible evolution of these lesions in CCA remain obscure. General inflammatory or proliferative and reactive processes were claimed to justify the possible carcinogenic evolution in Type I choledochal cysts, a form that alone represents the 80–90% of this class of diseases [85]. In a recent study, comparative transcriptome sequencing in Type Ia (cystic) and Type Ic (fusiform) choledochal cysts was performed [86]. Several genes involved in hormonal regulation and inflammation were identified, demonstrating an increased immune response in the fusiform type usually involved in more severe symptoms, including increased pancreatic enzymes and cholangitis [87]. Interestingly, gene expression of erythroblastic oncogene B (ERBB) 2 and wingless/integrated (WNT) 11 were up-regulated in fusiform and cystic Type I choledochal cysts, respectively. In fact, both of these molecular factors are thought to be involved in CCA development [88,89]. Despite the fact that this study presented some limitations, such as low patient numbers (*n* = 22), it has the merit to attempt to unravel the molecular mechanisms supporting Type I choledochal cysts and their possible evolution toward CCA. Another disorder classified in this chapter (Todani Type V), determined by a mutation in the polycystic kidney and hepatic disease 1 (PKHD1) fibrocystin gene and characterized by a frequent carcinogenic evolution, is represented by Caroli disease (CaD) [90]. In a mouse model of exon 4 PKHD1 disruption, intrahepatic biliary cysts and periportal fibrosis were observed, greatly resembling CaD disease and supporting the role of this gene in the onset of the disease [91]. Unfortunately, no study has evaluated the molecular processes leading from CaD to CCA. In this regard, a general mechanism linking inflammatory response and autocrine stimuli to cancer growth should be advocated [92]; however, increased degradation of basal laminar proteins, such as laminin and procollagen IV, has been observed in dilated ducts of human and experimental CaD [93]. Interestingly, in CaD specimens, carcinoma in situ was typically identified in ducts with poor laminar protein composition, possibly suggesting an increased chance of spreading early tumoral lesions in this disease.

### 3.4. Molecular Mechanisms Linking Risk Factors with a Suggested Role in CCA Onset

As reported in the corresponding paragraph, several factors have been reported to have an association (sometimes weak and controversial) with CCA. Liver cirrhosis is considered the main risk factor for HCC, while the risk of iCCA increased nearly 30 times in comparison to control [94]. Molecular mechanisms increasing liver cancer in cirrhosis are probably shared by both HCC and CCA. Regenerative nodules encountered in liver cirrhosis are sustained by ductular reaction, possibly by the contribution of progenitor cells [95]. The cellular components of ductular reaction are positive for both cholangiocyte and hepatocyte lineages (CK-19 and HepPar1, respectively) [96], suggesting a possible evolution during chronic inflammatory stress toward both cholangiocyte- or hepatocyte-derived cancer. In keeping with this view, the rare mixed form of hepato-cholangiocarcinoma, a tumor presenting histological hallmarks of both HCC and CCA, has been associated with liver cirrhosis in 50–80% of cases [97].Another point supporting similar carcinogenic mechanism for both HCC and CCA in cirrhosis is the frequent changes in p53 observed [98]. In fact, in approximately 7% of regenerative nodules in cirrhosis, an alteration of this tumor suppressor gene was observed, while a wild-type gene is usually conserved in NAFLD or chronic hepatitis. The association of p53 changes with HCC is well-known, but it should be recognized that a dysfunction of this gene is also observed in iCCA [99,100]. It is interesting to note that loss of p53 in mice gives origin to tumors with bilinear (HCC/CCA) transformation [101], and gene repression increases nestin-positive undifferentiated cells able to evolve to HCC or CCA according to activation of Wnt or Notch signaling, respectively [102]. Therefore, liver cirrhosis would be able to sustain both HCC and CCA starting from the p53 alterations in a regenerative nodule and cause oncogenic changes that may give origin to one tumor or the other.

NAFLD, NASH, obesity and type II diabetes are possible risk factors for CCA. Since these conditions are frequently overlapping in the clinic, the net carcinogenic effect of each single component is not easy to address [103]. In a recent study, liver tissue affected by NASH, but not NAFLD, was found in approximately 22% of patients undergoing surgery for iCCA [104]. This finding suggests that fatty liver-related inflammatory and molecular processes may lead to iCCA with still unveiled mechanisms.

## 4. Conclusions

CCA is a severe disease with very limited therapeutic options at advanced stages and, therefore, better understandings of CCA disease progression may offer insight into potential therapies. Excluding a minority of subjects developing this cancer with a background of specific risk factors (such as PSC, liver flukes and others), in the vast majority of patients, a predisposing condition may not be identified. Low prevalence and large heterogeneity of CCA do not allow researchers to clearly determine the sequence of molecular events leading to development and progression of this cancer. For example, the role of stem cells is currently under deep investigation in CCA since approximately 30% of tumoral mass has been demonstrated to be constitued by these cells [105]. However, while the rich representation of stem cells in CCA suggests their important role in this malignancy, the molecular mechanism of this contribution remains unknown, warranting further research.

Further, in vivo models have proven to be suboptimal in the quest to understand CCA progression and its mechanisms of action. These include carcinogen-induced CCA in rodents, xenogenic CCA cell transplants in nude mice and others, which only partially recapitulate tumorigenesis found in humans [106]. Improvement in research may come from new complex systems, such as organoids (three dimensional cell culture reproducing the tumor microenvironment) constituted by patient-derived cancer cells [107]. Moreover, the extensive exploration of miRNAs, such as miRNA-21,which has been demonstrated to be an important CCA prognostic marker in serum, also seems a promising approach [108]. Finally, collaboration between basic and clinical researchers and cooperation in large, well-designed clinical trials may be a good strategy to overcome the translational gaps observed in CCA therapies.

In this review, we report the more recent findings regarding the possible mechanisms of action leading from well-known risk factors to CCA. We believe activation of some of these pathways maybe unrelated to a specific risk factor and, thus, represent targets for therapeutic intervention of CCA. Specific animal models have been identified for liver flukes or PSC and should be considered, not only as systems to study these diseases, but also to evaluate pre-tumoral processes leading to CCA. It is expected that improved knowledge of risk factors and associated molecular mechanisms will significantly enhance our general comprehension of CCA carcinogenesis and enable the development of specific therapeutic strategies against this aggressive cancer.

## Authors contribution

L.C. and T.Z.: Original Draft Preparation & Editing; I.L., V.M., L.K., B.E., M.M., W.Z., C.W., K.S., S.C.: Review & Editing; S.S.G. and H.F.: Supervision, Funding Acquisition & Editing; G.A.: Conceptualization Funding Acquisition & Editing; L.B.: Conceptualization, Methodology, Original Draft Preparation & Editing. All authors have read and agreed to the published version of the manuscript.
